# Reimagining psychosis prevention: responding to the accessibility issues of At-Risk Mental State (ARMS) services through a selective public health approach

**DOI:** 10.1192/bjb.2024.112

**Published:** 2025-06

**Authors:** Luke Brown, Siân Lowri Griffiths

**Affiliations:** 1 Centre for Applied Psychology, School of Psychology, University of Birmingham, Birmingham, UK; 2 Black Country Partnership NHS Foundation Trust, Dudley, UK; 3 Institute of Mental Health, School of Psychology, University of Birmingham, Birmingham, UK

**Keywords:** Prevention, psychotic disorders/schizophrenia, mental health services, psychosocial interventions, at-risk mental states

## Abstract

At-Risk Mental State (ARMS) services aim to prevent the onset of first-episode psychosis (FEP) in those with specific clinical or genetic risk markers. In England, ARMS services are currently expanding, but the accessibility of this preventative approach remains questionable, especially for a subgroup of FEP patients and those from specific ethnic minority communities. This commentary outlines the key debates about why a complimentary approach to psychosis prevention is necessary, and gives details for an innovative public health strategy, drawing on existing research and health prevention theory.

At-Risk Mental State (ARMS) services^1^ continue to remain the most established approach to preventing psychosis in the Western world.^2^ According to the definition set out by the World Health Organization^3^ (Table 1), ARMS services are classified as indicative primary prevention,^7^ as they aim to stop the onset of first-episode psychosis (FEP) in those with specific clinical or genetic risk markers, also known as the ultra-high-risk criteria. In England, UK, ARMS services are expanding, with all regional Early Intervention in Psychosis teams expected to deliver psychosis prevention to 14- to 35-year-olds.^8,9^ Despite such advancements, there has been little debate about the suitability of ARMS services for all FEP patients.^10,11^

**Table 1:** World Health Organization's classification of preventive approaches for mental disorders^3^

Public health classification of prevention	Gordon's classification of prevention,^4^ modified by the US Institute of Medicine^5^
**Primary prevention** seeks to prevent the onset (incidence) of a disorder or illness.	
	*Universal primary prevention* is defined as those interventions that are targeted at the general public or a whole population group that has not been identified on the basis of increased risk.
	*Selective primary prevention* targets individuals or subgroups of the population whose risk of developing a mental disorder is significantly higher than average, as evidenced by biological, psychological or social risk factors.
	*I*n*dicated primary prevention* targets high-risk people who are identified as having minimal but detectable signs or symptoms foreshadowing mental disorder, or biological markers indicating predisposition for mental disorders, but who do not meet diagnostic criteria for the disorder at that time.
**Secondary prevention** seeks to lower the rate of established cases of the disorder or illness in the population (prevalence) through early detection and treatment of diagnosable diseases.	
**Tertiary prevention** includes interventions that reduce disability, enhance rehabilitation and prevent relapses and recurrences of the illness.	

Table adapted from Kirkbride et al^6^ and Fusar-Poli et al.^7^

## The challenges of ARMS prevention

There are two main reasons why ARMS services are criticised for being the sole approach to prevent psychosis. First, there remains a lack of clarity about the proportion of patients who benefit from ARMS clinics. It is estimated that about a third of patients experience no ARMS symptoms before the onset of psychosis, and so would be ineligible to access ARMS care even if they were to seek help during the prodromal phase.^12^ Furthermore, transition rates from ARMS services to FEP is low (8–17%),^13–16^ and so it remains unclear how sensitive the ARMS criteria is to those who truly are at risk. The second critique of ARMS services is their constrained appeal. Only a small proportion (4.1%) of patients presenting to psychiatric care with a diagnosis of FEP come via ARMS services. ARMS services are most likely to be accessed by patients who voluntarily seek help from the healthcare system,^17^ and are disproportionately underused by individuals from Black ethnic backgrounds (African, Caribbean and British)^18^ despite this group being at increased psychosis risk.^19,20^ This may be caused by cultural differences in help-seeking preferences;^17,18,21–23^ alternative beliefs about the causes of psychosis;^24,25^ or the result of a more acute form of psychosis onset, resulting in urgent, involuntary and coercive psychiatric treatment.^26,27^ Collectively, these points raise questions about the accessibility and sensitivity of the ARMS preventative model, which is further concerning given the National Health Service's (NHS) commitment to preventative healthcare and reducing health inequalities.^28^

## Public health approaches to psychosis prevention

Although there is greater recognition of the need for complementary approaches to ARMS services,^10,11,29–31^ rare is there a discussion about how this can be achieved.^4^ Selective and universal public health preventative approaches (Table 1) have the potential to overcome the limitations of the ARMS model, as preventative care is directly targeted at the general population, in what is referred to as ‘upstream’ working.^32^ These approaches are likely to be more accessible and have a wider reach, as they exist outside of the boundaries of the psychiatric care system. They are also more likely to be acceptable and therefore more appealing, as they offer care in less stigmatising, less coercive and more culturally attuned settings.

One of the overarching mechanisms by which selective or universal prevention could act to stop psychosis transition is by addressing the social factors that predispose healthy individuals to psychosis, known as social determinants. These determinants act at the individual, neighbourhood and environmental levels, comprising of factors like socioeconomic disadvantage, childhood adversity and trauma, migration, discrimination, neighbourhood socioeconomic disadvantage, social capital, social fragmentation, ethnic density and cannabis use.^6,10,33^ Public health interventions are effective in acting on the social determinants of psychosis.^6^ Despite this, there continues to remain a lack of evidence demonstrating the direct effect of public health interventions in reducing future psychosis incident rates in the real world, and no clear agreement about a model of service delivery.

## Future considerations

According to Frieden's^34^ Six Components Model, innovation is central to the effective design and implementation of any public health programme. Building on this premise, we outline our considerations for building a public health preventative strategy for FEP.

### Selective prevention

Rather than employing a universal strategy, we think there is greater utility and better use of resources by adopting a selective preventative approach. This public health model would aim to stop the development of new FEP cases from subpopulations at increased social risk. Individuals within these subgroups may be asymptomatic or display nonspecific symptoms of mental distress associated to the social risk factors they have been exposed to. We also believe this work should be children and young people specific, as the onset of psychosis is most common in youth.^35^

### Risk prediction–detection modelling

To identify at-risk individuals from within the general population, a new prediction–detection tool will be needed. Through an innovative, data-science-based approach, this tool could be mathematically modelled on existing FEP patients’ sociodemographic information and social determinant data. By using real-world metrics, the tool should be able to: (a) identify neighbourhoods and communities at high risk, in terms of their probabilistic likelihood of containing future psychosis cases; and (b) predict the demographic level characteristics of at-risk individuals within those neighbourhoods. The tool would therefore enable a place-based focus to risk prediction and detection, which would facilitate localised prevention planning. There are existing examples of data-driven tools that utilise either patient^36^ or social determinant^37^ data to predict and forecast psychosis cases in clinical and population contexts. Although these digital technologies are not specifically designed to aid selective prevention programmes for FEP, they do provide support for what is achievable in this space through their combination.

### Collaborative case identification

An effective preventive strategy will need to consider the mechanisms by which FEP prediction–detection technologies are used to find at-risk cases in the real world. In addressing some of the accessibility issues of ARMS services, selective prevention will need to go beyond the psychiatric care system and reach into the wider social institutions that children, young people and families interact. We therefore feel a localised and coordinated network of institutions across the health and social sector will be best positioned to identify at-risk individuals in the community. Religious; voluntary, community and social enterprise, education and social care services are some of the likely candidates for this network. We also believe there is a role for the NHS, particularly Child and Adolescent Mental Health Services^38^ and general practices, because of their specialised or localised focus on child and family health.

In a practical sense, the detection of at-risk cases would involve a whole range of integrated measures across the network of providers. For example, in the health and social care system, a nationally coordinated selective screening programme^39^ could be used to proactively invite at-risk individuals for routine mental health screening assessments. Outside of the NHS, voluntary, community and social enterprise organisations and schools in areas of high risk could be trained to spot early cases, leading to supported referral or screening processes.

### Multi-layered youth-focused preventative interventions

Existing evidence should be used to decide which preventative interventions are adopted within the selective prevention programme.^34^ Prevention will also need to be multi-layered, able to intervene on a range of direct and distal psychosocial developmental levels in childhood,^40^ and able to influence key social determinants.^6,41^

First, interventions should aim to address the impact of childhood adversity.^42,43^ Psychological interventions should be considered because of their effectiveness in targeting the effects of childhood abuse, neglect and victimization. For example, eye movement desensitization and reprocessing has been shown to reduce the symptoms of childhood trauma by adapting negative memory pathways and lessening one's reactivity to traumatic stimuli.^44,45^ Family-focused therapy should also be included, because of its effectiveness in addressing various adolescent mental health difficulties. Furthermore, eye movement desensitisation and reprocessing and family therapy have both been shown to lessen psychotic experiences in clinical and non-clinical populations.^46,47^

Second, a preventative strategy should also aim to address the effects of social disconnectedness, such as social fragmentation, social marginalisation and racial discrimination.^41,48^ Interventions that improve civic engagement should also be considered, including youth-focused social prescribing and educational/vocational participation schemes.^6^ At the neighbourhood level, improving community resources and infrastructure will also be pivotal. Cultural centres, community organisations, outdoor recreational areas and religious organisations are likely to act as protective factors,^41^ by providing greater community cohesion. Family interventions might lessen youth alienation, by improving family cohesion and connectedness.^49^

Finally, strategy should aim to lessen the impact of social economic disadvantage. Some examples might be improving the economic state of families in high-poverty neighbourhoods through direct payment schemes, which have been shown to reduce distress and anxiety in parents and children.^49^ Neighbourhood regeneration schemes that improve the physical quality of the built environment by planting trees, removing litter and landscaping vacant land should also be included,^49^ as these initiatives have been shown to lower depressive symptoms and improve self-worth amongst residents.

A placed-based health partnership^50^ will be most effective in delivering these interventions. For example, local authorities and public health departments could be responsible for delivering the community and neighbourhood-level components of the preventive strategy, whereas schools and social care organisations could be tasked to facilitate the individual and family level. This collaborative approach to prevention ensures that the most effective interventions are delivered at the right time and by the right provider.

In conclusion, the accessibility of existing preventative strategies for psychosis^51^ requires us to explore greater diversity in our approach.^11,38^ What is lacking is the how – the specific strategies that ensure that all communities have equal access to preventative care. We believe that a public health approach employing a selective preventative strategy offers a novel and equitable way to achieve this, by focusing on communities at increased risk in the general population and developing collaboration between the healthcare system and different social organisations. Interventions within such a strategy should be youth-focused and aim to target multiple levels within the life course of the young. Future pilot research is however needed to establish which preventive interventions have the greatest impact in reducing incident rates of psychosis in a population. From this, recommendations for health policy and political commitment can be generated, so that effective interventions can be expanded to the national stage.

## Data Availability

Data availability is not applicable to this article as no new data were created or analysed.

## About the authors

**Luke Brown,** BSc, MRes, PhD, ClinPsyD, is an assistant professor at the Centre for Applied Psychology, School of Psychology, University of Birmingham, Birmingham, UK; assistant professor at the Institute of Mental Health, School of Psychology, University of Birmingham, Birmingham, UK; and a principal clinical psychologist with the Early Intervention in Psychosis Service, Black Country Partnership NHS Foundation Trust, Dudley, UK. **Siân Lowri Griffiths**, BSc, MSc, PhD, is an assistant professor at the Institute of Mental Health, School of Psychology, University of Birmingham, Birmingham, UK.
